# Effect of Bear Garlic Addition on the Chemical Composition, Microbiological Quality, Antioxidant Capacity, and Degree of Proteolysis in Soft Rennet Cheeses Produced from Milk of Polish Red and Polish Holstein-Friesian Cows

**DOI:** 10.3390/molecules27248930

**Published:** 2022-12-15

**Authors:** Dorota Najgebauer-Lejko, Agnieszka Pluta-Kubica, Jacek Domagała, Katarzyna Turek, Iwona Duda, Jozef Golian

**Affiliations:** 1 Department of Animal Product Technology, University of Agriculture in Krakow, Balicka 122, 30-149 Krakow, Poland; 2 Department of Food Hygiene and Safety, Faculty of Biotechnology and Food Sciences, Slovak University of Agriculture in Nitra, Tr. A. Hlinku 2, 949 76 Nitra, Slovakia

**Keywords:** Polish Red cow, Polish Holstein-Friesian cow, bear garlic, soft cheese, physicochemical properties

## Abstract

This study aimed to assess the effect of milk source and bear garlic addition on the selected properties of soft rennet cheese. Cheeses were produced from cow milk derived from two sources: Polish Red cows (PR) and Polish Holstein-Friesian cows (PHF) with a 0.5% (*w*/*w*) addition of bear garlic (*Allium ursinum* L.) dried leaves. Chemical composition and fatty acid profiles (GC) were determined in fresh cheeses. Fresh and stored for two weeks cheeses were subjected to microbiological studies, i.e., total aerobic bacteria count (TABC); count of *Lactococcus* sp., yeast and molds; coliforms; analysis of the proteolysis extension by means of *o*-phthaldialdehyde (OPA) assay and free amino acids content (HPLC); antioxidant capacity as 2,2-diphenyl-1-picrylhydrazyl (DPPH) radical scavenging activity and ferric reducing antioxidant power (FRAP); as well as pH and water activity. Cheeses with bear garlic herbs were more prone to proteolysis but this was not accompanied by any effect on the microbial counts, water activity or pH. Cheeses produced from PR milk contained less monounsaturated fatty acids (MUFA) but were richer in n-3 PUFA and had a lower n-6/n-3 FA ratio than cheeses from PHF milk. Bear garlic addition increased DPPH anti-radical power but had less of an effect on the FRAP values.

## 1. Introduction

The most famous and valued cheeses in the world are produced locally from unpasteurized milk that often comes from local breeds of cattle, sheep, goat or other dairy animals kept on natural pastures in the unpolluted environment. The high and outstanding quality of these products is strongly linked to the specified region of origin. In the EU, such products may be promoted and protected by quality certification systems, such as PDO (Protected Designation of Origin), PGI (Protected Geographical Indication) or TSG (Traditional Speciality Guaranteed). Polish Red cattle (PR) is the oldest native cattle breed reared mainly in the mountain and sub-mountain areas of the southern part of Poland. PR cattle is now protected by the national Genetic Resources Conservation program, which involves 2419 milking cows in 259 herds (as for 2018). The milk of Polish Red cows is characterized by a high content of total solids, including protein and fat concentrations, a favorable protein-to-fat ratio, as well as a favorable protein and fatty acid profile when compared with milk derived from the most popular and high-yielding Holstein-Friesian cows. These features, together with a high calcium content and relatively short coagulation time, make this milk type an excellent raw material for cheese production. This is confirmed in the fact that milk from PR is the only cow milk type allowed for the production of Polish PDO cheeses (Bryndza Podhalańska, Oscypek, Redykołka) However, the production of the above-mentioned traditional high quality dairy products is only on a small scale and needs promotion and protection as it constitutes the tangible and intangible heritage of local society [1].

Herbs and spices are added to different cheeses, e.g., Cheddar, Gouda, Jack, Montaray Jack, Liptauer, Feta, Bouletted’Avesnes, Raclette, as well as different acid-type cheeses and processed cheeses, mainly in order to give attractive sensory properties, especially specific flavors. Fresh cheeses are the most common cheeses to be flavored with aromatic herbs. Plant materials may be added as ingredients during cheese production (e.g., mixed with milk or cheese curd) or used as a coating for a cheese or in some cases even used as clotting agents. The herbs and spices most frequently added to cheese comprise: peppers (red, black, green), thyme, cloves, cumin, parsley, paprika, onion and garlic. However, the results of many studies have revealed that plant extracts added to cheese may also play other important roles, acting as potent and natural antioxidants and preservatives able to inhibit the growth of pathogenic and spoilage microorganisms and to retard lipid oxidation. Supplemented cheeses may also demonstrate increased health-promoting activity due to the well-established therapeutic properties of the selected herbs [2,3,4,5,6].

Bear garlic (*Allium ursinum* L.) called also ramson, wild garlic, gypsy garlic, forest garlic or broad-leaved garlic is a wild edible plant belonging to the genus *Allium* of the *Amaryllidaceae* family occurring in Northern and Central Europe and North Asia. It is a perennial plant, with an underground bulb (onion), soft and short stem, oval leaves and white flower umbels blooming from April to June. It grows in nature in large colonies on a floor of wet mixed or deciduous forests or it can be cultivated (in Poland harvesting of bear garlic from a natural state is illegal). Bear garlic contains many bioactive substances, such as vitamins C and E, provitamin A, minerals (phosphorous, magnesium, potassium, iron, manganese, zinc, sulfur, selenium), glutamyl peptides, adenosine, and volatile oils, and shows strong antimicrobial properties [7,8]. *A. ursinum* is rich in various sulfur compounds, primarily glutamyl peptides and sulfoxides, such as S-alk(en)yl-L-cysteine, sulfoxides (methiin, alliin, isoalliin, ethiin, propiin) and their volatile derivatives thiosulfinates (allicin, methyl-allyl thiosulfinate, dimethyl thiosulfinate) as well as (poly)sulfides. The leaves of wild garlic contain also considerable amounts of polyphenolic compounds, mainly kaempferol derivatives, and other antioxidant substances such as carotenoids, glutathione, and the antioxidant enzymes catalase and glutathione peroxidase [9,10]. Bear garlic has been used for many years in folk medicine due to its antiseptic, antifungal, antibiotic, antioxidant and detoxifying properties. Therefore, it has been recommended as an immune system booster, digestive stimulant and in the treatment and prevention of many diseases, including cancers, cardiovascular, respiratory and rheumatic diseases and to combat skin disorders e.g., acne problems, as well as to support wound healing [8,10]. Chopped fresh or dried leaves, flowers and bulbs of this plant are also used as a seasoning and additive for meals and foodstuffs, e.g., meat, salads, soups, sauces, pasta, sandwiches. Liqueurs and wines may also be produced from the leaves or bulbs of *Allium ursinum*. Minced leaves are also added to many regional cheeses e.g., in Poland to Koryciński Cheese, Rennet Cheese with a Forest Note [11,12,13]. Bear garlic is also traditionally added to Tomme fermière à l’ail des ours, Tomme vaudoise à l’ail des ours cheeses produced in France and Switzerland. On the other hand, produced in UK Wild Garlic Yarg is wrapped in *Allium ursinum* leaves [3]. Traditional herby ripened (typically for 3 months) cheeses called “Otlu peynir” with a single or mixed herbs, including *Allium* sp., *Anthriscus nemorosa*, *Ferula* sp., *Prangos* sp., *Silene vulgaris*, *Thymus* sp., *Mentha* sp. are widely produced both in local family farms or in industrial dairy plants and are very popular among consumers in Turkey. The herbs are usually added in the amount of 2% to cheeses. These herbs not only impart flavor to the cheese but also may act as preservation agents because of their reported antimicrobial effects [14,15,16]. In the past, traditional herb cheese constituted also an important source of vitamins in diet of mountain villagers during winter periods under the conditions of a shortage of fresh vegetables [17].

Recently, some reports focusing on the effect of herb additions on the antioxidant capacity and or microbiological quality of different cheeses, including Mozarella cheese [18], Lor whey cheese [2] or Herby cheese [19] have been published. However, to the best of our knowledge no comprehensive study was devoted to soft rennet cheeses supplemented with bear garlic. In that light, the aim of the present study was to produce and compare unripened soft rennet cheeses produced from cow milk coming from two different cattle breeds, i.e., Polish Red and Polish Holstein-Friesian, with and without the addition of wild garlic, with regard to the basic chemical composition, profile of fatty acids, antioxidant properties as well as microbiological quality, and susceptibility to proteolysis during cold storage. An additional goal of the study was to develop a novel functional food product i.e., herbal cheese, relatively easy to produce, from milk coming from an autochthonous cattle breed to expand the range of products made from this raw material.

## 2. Results

### 2.1. Basic Chemical Composition and Fatty Acid Profile

Milk derived from Polish Red (PR) cows was characterized by higher dry matter content, including fat, protein, and ash concentrations than milk from Polish Holstein-Friesian (PHF) cows (Table 1).

Fresh cheeses produced from different milk sources were characterized by different total solids content, which was significantly higher in cheeses produced from milk of PHF cow breed (Table 2). However, it was accompanied by only slightly and insignificantly (*p* > 0.05) higher concentrations of fat, protein, and sodium chloride in PHF cheese when compared to PR cheese. In turn, an addition of 0.5% bear garlic herb increased total solids and protein contents in soft rennet cheeses. The ash, fat and salt concentrations were not affected by the bear garlic—supplementation.

Cheese produced from PR milk when compared to PHF was characterized by a higher content of saturated fatty acids (SFA), including caproic (C6:0), caprylic (C8:0), capric (C10:0), lauric (C12:0), tridecylic (C13:0), myristic (C14:0) SFAs, but lower content of stearic C18:0 FA (Table 3). Significantly lower contents of monounsaturated fatty acids (MUFA), especially oleic (C18:1 (n-9)) FA, slightly higher decanoic (C10:1) FA and higher cis-vaccenic (C18:1 (cis-11)) FAs were also detected in PR cheeses. On the other hand, cheeses from PR milk were more abundant in PUFA, including linolelaidic (C18:2 (trans-9,12)), alpha-linolenic (C18:3 (n-3); ALA) FAs. However, they contained lower concentrations of linoleic (C18:2 (n-6)) FA. Cheeses produced from both milk sources did not differ with regard to conjugated linoleic (CLA) and gamma-linolenic acid (C18:3 (n-6); GLA) levels. Cheeses produced from PR milk contained a lower proportion of unsaturated to saturated FAs than cheeses from PHF milk. Moreover, the content of monounsaturated fatty acids (MUFA) was about 7% higher in PHF cheeses. However, higher percentages of PUFA (3.1–3.5% vs. 2.6–2.7%) were determined in PR cheeses. The prevailing SFA in both cheeses was C16:0 (palmitic FA), followed by C18:0 (stearic FA) present in the 50% lower amount and myristic FA (C14:0). MUFA in the analyzed cheeses were represented in the greatest amount by the oleic acid (C18:1), which constituted on average 21–32% of all FA in cheese fat and 69–73% and 81–83% of MUFA in PR and PHF cheeses, respectively. Among PUFA, linoleic acid (LA) was present in the highest amount. The addition of bear garlic to the cheeses had little effect on the FA profile. Only in the herby PR cheeses was a significantly increased concentration of C14:1 MUFA noticed (*p ≤* 0.05). Significant interactions of both studied variability factors found for the concentration of myristoleic acid confirmed that the herbal addition differently influenced its level in PR and PHF cheeses.

### 2.2. Microbiological Quality

The total aerobic bacteria plate count (TABC) of microorganisms in the analyzed cheeses was in the range of 10^8^–10^9^ cfu/g (Table 4). This number was slightly higher in cheeses produced from milk derived from Polish Red cows when compared with cheeses from milk from PHF cows. TABC slightly decreased during two-week storage, especially in plain cheeses, but generally was not affected by the bear garlic addition. However, a significant interaction was found between cheese type and storage time for TABC as total number of bacteria tended to decrease more in plain cheeses, and was almost unchanged in herbal treatments (statistical effects in Table 4). *Lactococcus* sp. bacteria constituted on average 97% and 94% of TABC in cheeses produced from PR and PHF cow milk, respectively. Analyzed cheeses contained yeasts and molds but no coliforms were present in the analyzed samples. The number of contaminating microorganisms was not affected by any of the studied factors. No growth of yeasts was observed in the fresh cheeses produced from PR milk, in the other cheese samples they were present in the amount of up to 4.22 log cfu/g (Table 4). Molds were present only in some samples of cheeses produced form PHF milk in the average number of 0.94–1.05 cfu/g without any effect of bear garlic or time of storage. Water activity and pH of cheeses turned out to be not affected by source of milk or bear garlic addition and did not undergo any significant changes during two weeks of cold storage.

### 2.3. Proteolysis

The results of ANOVA revealed that the source of the milk used for cheese production had a significant effect on the degree of proteolysis expressed in OPA values and levels of free amino acids (FAA), including all FAA except for Pro and Phe (Table 5, Table S1 Supplementary Material). Higher values of total FAA, OPA and the above-mentioned specific FAA were detected in cheeses produced from PHF milk. There were also significant interactions between milk source and cheese type in case of total FAAs, and all specific FAAs except for Glu, Arg, Pro, Tyr, and OPA value (Table S1). On the other hand, free amino acid Cys was only detected in cheese derived from PR cow milk. The bear garlic addition also influenced FAA and OPA, with the exception of the concentration of free Thr and Pro. These effects were more pronounced in cheeses produced from PHF milk, which were characterized by significantly higher concentrations of total FAAs, including Leu, Ile, Lys, Met, Val, His, Ser, Asp when supplemented with herbs (Table S1). On the contrary, in cheeses produced from PR milk bear garlic addition influenced significantly higher contents of only Phe and Ile. Moreover, herbal cheeses from PHF milk were more prone to an increase in proteolysis degree during two-week cold storage. However, storage time turned out to be a significant factor for the lower level of FAA, including Gly, Thr, Pro, Phe, Cys, as well as total FAA and OPA value (the least square means from ANOVA). The only statistically significant changes in the concentration of selected FAA during storage were noticed in case of Gly, in herbal cheese from PHF milk, and Cys in herbal cheese from PR milk.

Pro was the predominant FAA in all cheese samples produced from PR milk, constituting approximately 15–18% in stored cheeses to 29–30% in fresh cheeses of the total FAAs content. On the contrary, in cheese extracts from PHF milk, the Pro concentration was similar in PR cheeses but the most abundant FAA was Glu (on average 19% in herbal cheeses, 26–28% in plain cheeses). Except for these, two FAA cheese samples contained also considerable amounts (more than 10 mg/kg) of free Lys, Thr, Tyr, Asp (N-PR, fresh) and Ala (N-PR, stored) and Leu, Val, Phe, Ile (BG-PR, fresh) as well as His, Arg, Met (BG-PR, stored). In cheeses from PHF milk, the only FAA present in the amount below 10 mg/kg were Ser, Gly, Hist, Met, (in N-PHF stored samples) and additionally Ile, Arg, (N-PHF, fresh). In herbal cheese made from PHF cow milk, only Gly in fresh samples was detected in a concentration below 10 mg/kg and Cys was not detected (as in all PHF cheese samples).

### 2.4. Antioxidant Activity

Table 6 presents results of the total phenolic content (TPC) and antioxidant activity against 2,2-diphenyl-1-picrylhydrazyl (DPPH) radical and ferric reducing antioxidant power (FRAP).

Generally, higher values of TPC and antioxidant activity were obtained for cheeses supplemented with herbs (BG) when compared to the plain treatments, but differences were not statistically significant. The only statistically significant difference in the total phenolic content (TPC) was observed between fresh plain cheeses and stored herbal cheeses produced from PHF milk. Another significant difference was noted in the anti-radical power (ARP) between fresh natural cheese from PR milk and herbal cheeses from PHF milk (fresh) and PR milk (stored). A significant interaction was found between milk source and storage time regarding ARP value. For cheeses produced from PR milk, radical scavenging capacity increased during storage and did not change after two-week storage for the PHF cheeses. The highest scavenging activity against DPPH radical was observed for stored herbal cheese from PR milk.

## 3. Discussion

Higher dry matter content, including fat, protein and ash concentrations determined in raw milk derived from Polish Red (PR) cows, when compared to milk from Polish Holstein-Friesian (PHF) cows, followed the general rule that cows of autochthonous breeds produce more concentrated milk than cows of specialized breeds [20]. This is also in agreement with data from the literature indicating that Polish Red cows deliver milk with a relatively high content of protein and fat, which make this milk type especially suitable for cheese production [1]. Most of the obtained data for PR milk values (total solids, fat and protein concentrations) were consistent with the ranges reported by Litwińczuk et al. [21], i.e., 13.30 ± 1.02% TS, 4.35 ± 0.73% fat, 3.61 ± 0.50% protein, whereas PHF milk was characterized by slightly lower TS and protein content than values reported for Polish Holstein-Friesian cows kept in the intensive system. Lactose content in milk was lower than those reported in the literature values. Higher fat content in PR milk suggests that the cows were fed a diet richer in fiber, which promotes increased lipids concentration in milk [20]. Regarding cheese chemical composition (given in Table 2), we hypothesize that the increased total solids and protein contents in herbal cheese treatments could be the result of more intense curd processing during mixing of the fresh curd with herbs, which promoted whey release from the curd.

According to data in the literature, milk obtained from PR cows, when compared to PHF cows, has a lower SFA content (61% vs. 67%), higher concentrations of MUFA (33% vs. 30%) and PUFA (5% vs. 3%) and a even seven-times higher content of CLA [1]. In our study, cheeses obtained from milk derived from PR cows were characterized by the higher SFA, lower MUFA and similar CLA contents than cheeses manufactured from PHF milk. On the other hand, these cheese treatments were above two-times richer in ALA as well as having higher concentrations of PUFA and n-3 FA, known to possess health-promoting properties. This is in agreement with the study of Bonanno et al. [20], who reported higher ALA, PUFA and n-3 FA in cheeses produced from cow milk coming from an extensive farming system and animals fed more green forages when compared to an intensive farm system with a smaller proportion of natural pasture and higher levels of hay in the animal diet.

Moreover, a much lower n-6 to n-3 FA ratio was calculated for cheeses from PR milk when compared to cheeses produced from PHF cow milk (Table 3). According to scientific data, a balanced n-6/n-3 FA ratio in a diet is an essential factor for human health as a high intake of n-6 FA and a high ratio of n-6/n-3 are associated with obesity, increased lepton and insulin resistance, and hyperactivity of the endocannabinoid subsystem. On the contrary, physical activity and diet characterized by the lower i.e., 1–2/1 n-6/n-3 FA ratio may help in prevention of obesity [22]. In that context, our cheeses produced from milk coming from PR cows were characterized by the more preferable proportion of n-6/n-3 FA, with a higher content of n-3 ALA and a lower content of n-6 LA than PHF cheeses.

According to Sagdiç et al. [15], the final number of microorganisms in herby cheeses may depend on the salt concentration, pH value, as well as the type and amounts of herbs added. In traditional cheeses produced without milk pasteurization, the authors reported contamination with coliforms, also psychrotrophic, lipolytic and proteolytic bacteria, *S. aureus*, yeasts and molds were present in cheeses produced from raw milk.

Znamirowska et al. [23] reported that the addition of 1% bear garlic powder (obtained from freeze-dried leaves) to sheep milk slowed down the fermentation during kefir production and resulted in a slightly decreased acidity of the final products. This suggests that bear garlic may have an inhibiting effect on the growth of lactic acid bacteria (LAB). On the contrary, there are also some reports indicating that plants from the *Allium* genera may improve the viability of lactobacilli in cheeses, for instance Mehdizadeh et al. [24] observed better survival of probiotic *Lb. acidophilus* (PTCC 1643) in white cheeses upon addition of *Allium ampeloprasum*. On the contrary, no positive or negative impact of the dried bear garlic addition to cheeses on the count of LAB were observed in our study.

Coskun [25] reported that herbs, including wild *Allium* sp., applied during cheese production delayed, but did not fully cease, the growth of coliforms in a traditional herby cheese. The prevention of coliform contamination and the extension of the shelf life of cheese upon wild garlic addition were also confirmed in a study by Gliguem et al. [26] that evaluated the effect of *Allium roseum* leaves supplementation on double cream cheese quality. As bear garlic is known for its strong antifungal activity, mainly due to the high content of allicin [23], it can be also expected that the growth of yeasts and molds during cheese storage should be reduced upon its supplementation. Data presented in Table 4 show that the number of yeast cells was lower in herbal cheeses, but the differences compared to plain cheeses turned out to be insignificant from a statistical point of view. In study by Ivanova et al. [27]), extracts of bear garlic were effective inhibitors of *Staphylococcus aureus* but did not show any inhibition effect against *Escherichia coli* and moderate antifungal activity against *Candida albicans*. The authors stressed that the main antimicrobial components of wild garlic are organosulfur compounds which are relatively unstable. Dżugan et al. [28] also reported the antifungal and antibacterial action of *Allium ursinum* extracts, especially against *Aspergillus niger, Candida albicans*, *Saccharomyces cerevisiae, Enterococcus rhamnosus, Salmonella enteritidis, Brochothrix thermosphacta* but the authors highlighted that heat treatment results in a considerable decrease of this activity. This indicates that air-drying of the bear garlic leaves, applied also in the present study, may reduce their antimicrobial activity.

The dried leaves of bear garlic itself, used in our study, contained on average 4.30 log cfu/g TABC and 4.24 log cfu/g lactic acid bacteria, and did not contain yeasts, molds or coliforms in 0.1 g. Stanisavljević et al. [29] reported that *A. ursinum* contained a considerable amount of lactic acid bacteria (LAB), including *Streptococcus, Lactobacillus* and *Bacillus* genera and some of the isolated strains (*L. fermentum*) exhibited probiotic potential. Although the bear garlic used in our study also contained a considerable amount of LAB (~10^4^ cfu/g) it seems that this did not affect the concentrations of LAB in the final products.

Proteolysis in the production and storage of cheese may have both desirable and undesirable effects. It may result from the action of chymosin from rennet, indigenous proteinases from milk (e.g., plasmin) and proteolytic enzymes produced by the starter and non-starter cultures of microorganisms (bacteria, yeasts and molds) during cheese manufacturing and ripening. On the other hand, proteolysis may negatively affect cheese quality during storage when it is derived from the action of heat-resistant proteinases produced by psychrotrophic microorganisms or alkaline milk proteinase [14,30]. Therefore, factors such as the quality of raw milk, type of coagulation enzyme, type of starter bacteria e.g., autochthonous in raw milk or different starters in pasteurized milk, hygienic standards, and conditions on ripening and storage may significantly influence the degree and routes of proteolysis in cheese. Other factors which may influence the degree of proteolysis in cheeses include, e.g., salt concentration and pH [31].

Results of the free amino acids (FAAs) analysis shown in Table 5 provide detailed information about proteolytic changes in the cheese samples. Generally, it was proposed that hydrolysis of proteins in rennet cheeses such as Gouda-type cheese, starts by the action of chymosin on α_s1_-casein to produce peptides which are further degraded by LAB protease and aminopeptidase into smaller peptides and free amino acids [32]. The levels and compositions of the products of protein degradation, such as peptides and FAA may be an important feature as they have a great influence on the cheese flavor, appearance, and texture, and some may exhibit antioxidant activity [31,32,33,34]. For instance, Wallace and Fox [35] reported that FAA such as Glu, Met, Leu, have a great impact on flavor development in Cheddar cheese. According to the reports of Mangia et al. [36] and Wallace and Fox [35] Pro is the most abundant FAA in fresh cheese, such as Fiore Sardo and Cheddar, but its content decreases during cheese ripening. In turn, other FAAs, such as Phe, Glu, Leu, Val, Ile and Lys may become the prevailing FAAs during cheese ripening [37]. However, the content of some FAAs may also further decrease due to utilization by the mesophilic lactobacilli [37]. Bearing in mind that our cheeses were not subjected to ripening but cold stored for a limited time of two weeks, these findings are partially consistent with the results of our study, as Pro was the main FAA in PR cheeses and in PHF cheeses, Glu dominated, indicating, together with the higher total FAA and OPA values, that these types of milk-produced cheeses are more prone to protein degradation. According to Mangia et al. [36], the high content of Pro may suggest a substantial hydrolysis of the β-casein in cheese.

According to Coşkun and Tunçtürk [14], wild garlic additions in the pickled form to cheese grains promoted proteolysis and lipolysis in rennet-ripened herby cheese. The authors proposed that this phenomenon may be due to the action of natural microbiota, e.g., yeast and molds, present in pickled herbs. In the study by the above-mentioned authors, water-soluble N, TCA-soluble N, and PTA-soluble N as indicators of proteolysis degrees were affected significantly (*p* ≤ 0.05) by increasing herb additions to cheese (from 0 to 3%). The positive and content-dependent effect of herb additions on the degree of protein degradation in Turkish Otlu (herby) cheese was also reported by Tarakci and Temiz [38]. Generally, these observations are partially consistent with the results of our study, however, the microbiological evaluation indicated that added dried herbs did not affect the count of microorganisms, including yeast and molds, in cheeses.

Among different parameters, the concentration of salt (NaCl) is the factor reported to influence pH, water activity, microbial growth, and degree of proteolysis in cheeses (Rulikowska et al., 2013). The content of sodium chloride in the analyzed herein cheeses was in the range of 0.4–0.6%, and this corresponded with the lowest salt concentration in Cheddar studied by the Rulikowska et al. [38] which, when compared to higher saltiness (up to 3%), reduced pH and buffering capacity and increased water activity and the growth of LAB, and enhanced proteolysis. According to these findings, the relatively low salt concentration in our cheeses should not have any mitigating effect on the microbial growth and rate of protein hydrolysis.

Generally, large deviations were obtained between result series for antioxidant properties of cheese samples, which resulted in the large standard deviations calculated for the results obtained from the two series of produced cheeses. The reason for these findings may result from the instability of antioxidant compounds in the analyzed cheeses, the small addition of herbs to cheeses (0.5%) or the nature of specific antioxidant substances in bear garlic which may not have sufficiently reacted in the applied assays. Antioxidant properties (scavenging effect against superoxide and hydroxyl radicals) of the extracts obtained from different organs of *Allium ursinum* plant, including leaves, resulted from both the presence of antioxidants such as flavonoids, glutathione, carotenoids, chlorophyls, vitamin C, and high activity of antioxidant enzymes including catalase and peroxidase [10,28]. Due to the high content of antioxidant substances, herbs added to cheese are expected to increase the antioxidant capacity of the final product. For instance, in the study by Kose and Ocak [19], herbs such as *Allium vineale* L., *Chaerophyllum macropodum* Boiss., *Ferula rigidula* DC., increased total phenolic concentration and antioxidant activity measured against DPPH radical of herby sheep cheese. There are many assays applied for measurement of the antioxidant capacity of food stuffs and herbs, and the results of many studies have indicated that the measured antioxidant activity is highly dependent on the method used for its evaluation. For instance, Çelik et al. [17] reported that FRAP (ferric reducing antioxidant power) turned to be non-respective to thiol-type compounds, therefore, sulfur-containing antioxidants present in bear garlic (*Allium ursinum*) do not react efficiently in this assay. Moreover, the volatile oil of bear garlic poorly reacted as DPPH and ABTS radical scavengers, whereas it showed a strong antioxidant effect in the beta-carotene-linoleic bleaching assay [39]. On the contrary, Lachowicz et al. [7] determined a considerable amount of total phenolic content (TPC) and high antioxidant activity measured using ABTS, DPPH and FRAP methods in different morphological parts, especially in the leaves, of bear garlic. Generally, there has been great diversity between the results reported by different authors, and this discrepancy may be due to many factors connected with the characteristics of the raw material, e.g., variety, stage of maturity, climatic conditions, agricultural practices, time and storage conditions after harvest, and/or with extraction procedure (including type of extraction solvent) and applied methods of analyses [7,19,29,40].

Additionally in cheeses the antioxidant capacity of bear garlic constituents may be altered by interaction with components of cheese, e.g., with milk proteins which have a great affinity to bind polyphenols [41]. Also, the salting of cheese may lower the content of measured antioxidant phenolic compounds [19].

## 4. Materials and Methods

### 4.1. Materials

Milk for cheese production was purchased from two sources (individual, small milk farms), i.e., (1) milk farm KHiNO “Polan” Sp. z o.o. from Dziekanowice (near Krakow, Poland)—milk from Polish Holstein-Friesian *Black*-and-*White* cows (PHF) and (2) milk from Polish Red cows (PR) delivered by Dairy Cooperative in Bochnia. Both milk types were obtained in the same region (Lesser Poland Province) and milk was delivered in the same season of the year (June and September). The basic chemical composition of both milk types determined in this study according to the respective methods is provided in Table 1.

Mesophilic, aromatic culture FD-DVS CHN-19 (Chr. Hansen, Denmark) consisting of *Lactococcus lactis* subsp. *cremoris*, *Leuconostoc* sp., *Lactococcus lactis* subsp. *lactis*, *Lactococcus lactis* subsp. *lactis* var. *diacetylactis* was used as a starter culture in cheese production. Microbiological rennet Fromase 2200 TL (DSM, Delft, The Netherlands, 2250 IMCU/g) was used as coagulant.

Leaves of bear garlic (*Allium ursinum* L.) were collected from the private plot in the spring period in Ropa—a village located in Gorlice County (Lesser Poland Voivodeship), air-dried in the oven at the laboratory of University of Agriculture in Krakow and stored in a glass jar in dark, dry place and chopped with a knife directly before usage for cheese supplementation.

Microbiological media, i.e., PCA, M17, chloramphenicol and VRBL agars and buffered peptone water were purchased from BioMaxima (Lublin, Poland). Folin-Ciocalteu’s phenol reagent, Trolox and gallic acid monohydrate were purchased from Fluka (Buchs, Switzerland; Copenhagen, Denmark and Madrid, Spain), 2,2-diphenyl-1-picrylhydrazyl (DPPH), and 2,4,6-tris(2-pyridyl)-*s*-triazine (TPTZ) from Sigma–Aldrich (Steinheim, Germany and Buchs, Switzerland). All other chemicals used were of analytical-reagent grade or HPLC-grade.

### 4.2. Cheese Production

**Figure 1:**
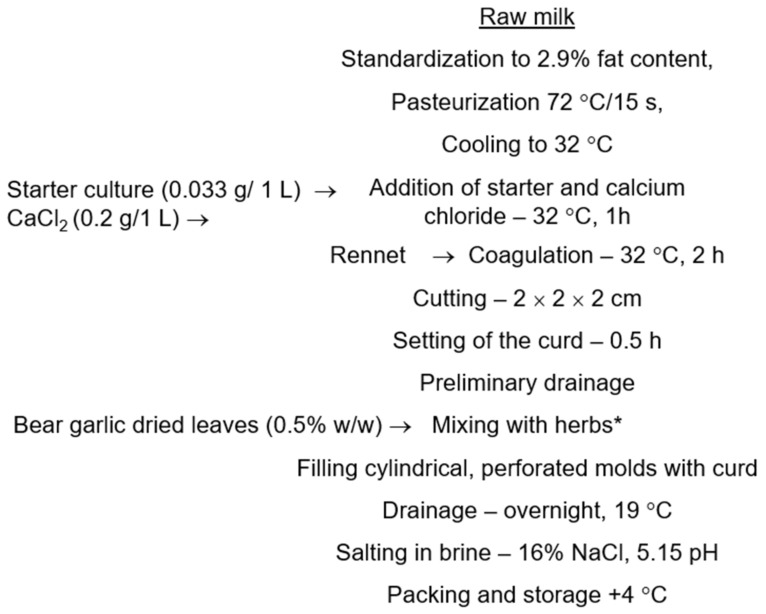
Cheese production procedure (* this stage was omitted during production of the control/natural cheese without herbs).

Soft rennet cheeses were produced at the Department of Animal Product Technology of the University of Agriculture in Krakow (Poland) following the procedure shown in Figure 1. Fresh, raw milk (20 L) was filtered through the cotton cloth, warmed to the centrifugation temperature of 40–45 °C, centrifuged to separate fat, standardized to 2.9% fat concentration, and pasteurized at 72 °C for 15 s in a mini cheese kettle equipped with a stirring shovel (Plevnik d.o.o., Dobrova, Slovenia). After cooling to 32 °C calcium chloride dissolved in small amount of cold water and starter culture were added to milk and milk was gently mixed for an hour before adding rennet solution. The necessary amount of 1% (*w/v*) rennet solution was calculated to obtain firm curd after 2 h of coagulation. After completing all production steps shown in Figure 1 singular cheeses (approx. 150 g cylinders) were wrapped in polyethylene cling film and stored in a refrigerator at 4 °C.

### 4.3. Methods

#### 4.3.1. Basic Chemical Composition

The basic chemical composition of cheese samples was determined by the respective AOAC [42] and ISO [43,44] methods: moisture content was determined by loss-on-drying in an air oven at 102 ± 1 °C, total protein content by Kjeldahl method using Büchii Digestion and Distillation Units (K-345 and B-324; Büchii, Switzerland), fat content by the Van Gulik method, chloride content by potentiometric titration method, and ash content in a muffle furnace at 550 °C.

#### 4.3.2. Determination of Fatty Acids Profile in Cheese

The determination of the fatty acids profile of cheese samples was performed by means of gas chromatography. Fat was extracted from cheese samples according to the procedure described by Folch et al. [45]. Subsequently, the extracted fat was subjected to esterification using the method reported by Ledoux et al. [46].

A uniform sample of thoroughly blended cheese (1 g) was homogenized for 10 min with 15 mL of 2:1 (vol.) ratio of chloroform: methanol mixture. After leaving for 5 min, homogenization was repeated for another 5 min. Obtained homogenate was filtered through paper filter and the volume was adjusted to 15 mL with 2:1 chloroform: methanol mixture. Then 3 mL of 0.74% KCl was added and the mixture was shaken and left for phase separation. After discarding upper water-alcoholic phase, lower chloroform phase was dehydrated using anhydrous sodium sulfate and dried under a current of nitrogen at 45 °C.

The extracted cheese fat was subjected to esterification. Briefly, 5 mg of fat was dissolved in 50 μL of toluene and 100 μL of 2 M sodium methoxide solution at ambient temperature for 20 min. Subsequently 0.5 mL of 14% methanolic solution of boron trifluoride was added to the mixture and left for another 20 min. Methyl esters of fatty acids were extracted twice using 2 mL of hexane. Prepared in such a way samples were injected in the amount of 1 μL into the BPX-70 chromatographic column (60 m × 0.25 mm × 0.20 mm) of the TRACE 1300 chromatograph (Thermo Scientific, Waltham, MA, USA) equipped with a flame ionization detector (FID). Helium was used as carrier gas (5 mL/min). Split flow was set as 10 mL/min, FID detector temperature was 250 °C, dispenser temperature was 220 °C. The analysis was carried out using the following temperature program: from 60 °C (holding for 3 min) to 200 °C with temperature growth rate: 7 °C/min, holding at 200 °C for 20 min. Fatty acids were identified by comparing the retention times of FA methyl esters (FAME) with their standard mixture: Supelco 37 FAME Mix and PUFA No.2 (Animal Source) (Sigma-Aldich Co., St Louis, MO, USA). The amounts of individual FAs (FA profile) were estimated on the basis of the identification and calculation of the relative peak areas.

#### 4.3.3. Antioxidant Activity

Cheese samples for determination of total phenolic content (TPC) and ferric-reducing antioxidant power (FRAP) were subjected to extraction procedure adapted from Moldovan et al. [47]. Briefly, 1 g of the ground in a ceramic mortar cheese sample was weighted into the 15 mL plastic conical Falcon-type tube and adjusted to 10 g with 60% ethanol. Subsequently the mixture was mixed using vortex (800 rpm) for 30 min at ambient temperature. Then samples were centrifuged at 5000 rpm (2683× *g*) for 15 min at 4 °C using MPW-352 Centrifuge (MPW Med. Instruments, Warszawa, Poland). After removal of fat layer, clear supernatant was used for TPC and FRAP analyses.

- Total phenolic content (TPC)

Total phenolic content (TPC) in cheese extracts was determined by the Folin–Ciocalteu method. In this method, 0.1 mL of ethanolic cheese extracts were transferred into 25 mL glass graduated cylinders, followed by the addition of 7.9 mL of distilled water and 0.5 mL of 2 N Folin–Ciocalteu reagent. The contents of the cylinder were mixed, and after exactly 5 min 1.5 mL of 20% sodium carbonate aqueous solution was added. After shaking, the mixture was left to react for 2 h at an ambient temperature in a dark place. The absorbance of the mixture was read at a wavelength of 765 nm against blank sample with the use of a Helios Gamma spectrophotometer (Thermo Electron Corporation, Cambridge, England). The results expressed in gallic acid equivalents (mg GAE) were read from a calibration curve and calculated as per 100 g of cheese sample.

- Ferric reducing antioxidant power (FRAP)

Cheese ethanolic extracts (0.4 mL) were mixed with 3.6 mL of freshly prepared TPTZ reagent solution (10:1:1 mixture of acetic buffer—300 mM (pH 3.6); TPTZ (2,4,6-Tris(2-pyridyl)-s-*triazine*)—8 mM in 30 mM HCl and 20 mM FeCl_3_ aqueous solution) in test tubes. Samples were incubated for 10 min at 37 °C and then without any delay centrifuged using MPW-331 Centrifuge (2 min, 4000 rpm, MPW Med. Instrument, Poland). Supernatant was used for measurement of absorbance at 593 nm using Helios Gamma spectrophotometer (Thermo Electron Corporation, Cambridge, UK). Antioxidant potential measured as the ability to reduce Fe^3+^ to Fe^2+^ was expressed as mM Fe^2+^/100 g of cheese sample using the calibration curve prepared for the solutions of FeSO_4_·7H_2_O (0; 0.05; 0.1; 0.2; 0.3; 0.4; 0.5; 0.6 mM/L).

- Scavenging activity against DPPH radical

Radical scavenging activity against DPPH (2,2-diphenyl-1-picrylhydrazyl) of cheese samples was determined according to procedure described in the work of Najgebauer-Lejko et al. [41]. Briefly, 0.10 g of cheese sample and standard Trolox solution (0.1875 g in 1 L methanol) was vortexed and then incubated with 3.9 mL DPPH methanolic solution (0.028 g/L) for 2 h in a dark place at ambient temperature. Subsequently, samples were centrifuged (1400× *g*, 10 min) and absorbance of clear supernatant read at 515 nm against blank sample (0.10 g methanol). The amount of sample required to decrease the DPPH radical concentration by 50% (EC_50_) was calculated. Results were expressed as ARP (anti radical power), the reciprocal of EC_50_, in mM TE (trolox equivalent) per 100 kg of cheese sample.

#### 4.3.4. Microbiological Study, pH and Water Activity

For microbiological testing, ten grams of cheese were homogenized with 90 mL of sterile buffered peptone water and consecutive decimal dilutions were prepared by mixing 1 mL of preceding dilution with 9 mL of buffered peptone water (BioMaxima, Warsaw, Poland) in test tubes [48]. Total aerobic bacteria count (TABC) [49], number of mesophilic lactococci [50], yeast and molds [51], and coliform [52] were determined according to the respective standard procedures on the respective media, i.e., plate count agar (PCA), M17 agar, chloramphenicol agar and VRBL agar (BioMaxima, Poland). The plates were incubated under aerobic conditions at 30 °C for 72 h (TABC and *Lactococcus* sp.), 25 °C for 120 h (yeasts and molds) and 30 °C for 24 h (coliforms). After incubation, microorganisms were counted, and results calculated and expressed in log cfu/g.

Emulsions of cheese samples prepared in water (1:1) were subjected to pH measurement using a CP-411 pH-meter (Elmetron, Zabrze, Poland). The water activity of freshly grated cheese samples was measured using LabMaster-aw (Novasina AG, Lachen, Switzerland) [53].

#### 4.3.5. Proteolysis

The degree of proteolysis in fresh and stored cheese samples was measured using two methods, i.e., *o*-phthaldialdehyde spectrophotometric assay (OPA) and free amino acids (FAA) content determined by HPLC in cheese extracts. Cheese sample (12.50 g) was homogenized with 50 mL of warm distilled water (~40 °C) by grinding in a mortar and pestle. Cheese emulsion was transferred quantitatively to a volumetric flask washing with small portions of distilled water. After the addition 3–4 drops of 40% formaldehyde, to stop further proteolysis, mixing and cooling to 20 °C, the contents of the flask were adjusted to the volume of 250 mL. The mixture was shaken for 5 min using a laboratory shaker and left for 10 min at ambient temperature. Shaking was repeated at intervals of 10 min for approximately 1 h, and after that time the samples were placed in a refrigerator (~6 °C). After removal of the crystallized fat, 100 mL of liquid was collected with a pipette and filtered through a paper filter. The collected water extract was subjected to the OPA (*o*-phthaldialdehyde) and free amino acids (FAA) assays.

- Free amino acids (FAA) analysis by HPLC method

Water extracts of cheese samples (5 mL) were mixed with 5 mL of 40% trichloroacetic acid solution, vortexed for 10 min and centrifuged at 12,000× *g* for 10 min. Aliquots of 2 mL were dried under the nitrogen current and the residue was dissolved in 1 mL of 20 mM HCl solution.

Extracts were subjected to the determination of free amino acids concentrations by the reverse phase HPLC method using ACCQ Tag kit (Waters Corporation, Milford, MA, USA). For this purpose, 10 µL of the analyzed amino acid solution or standard was mixed with 70 μL of borate buffer (pH 8.2–9.0) and 20 μL of 6-aminoquinolyl-N-hydroxysuccinimidylcarbamate (AQC) in an acetonitrile solution (3 mg/mL). Separation was performed using Dionex Ultimate 3000 chromatograph (Thermo Scientific, Waltham, MA, USA) equipped with LPG—3400 SD gradient, four-channel pump, WPS 3000 TSL autosampler and FLD 3400RS four-channel fluorescence detector. Nova–Pak C 18, 4 μm, (150 × 3.9 mm) column (Waters Corporation, Milford, MA, USA) was used for analysis. Separation was carried out at 37 °C in two-components gradient set at 1 mL/min following the elution scheme: (A): acetate: phosphate buffer, (B): acetonitrile: water 60:40. The quantitative analysis was done using 1-point calibration with the analytical standards (100 pmol). Results were analyzed using the Chromeleon 7.0 Chromatography Data System software.

- OPA assay

The OPA (*o*-phthaldialdehyde) reagent was prepared daily (directly before assay) from the components and by the procedure described by Church et al. [30]. Sample solution in the amount of 100 μL was added to 3 mL of OPA reagent in a quartz cuvette, mixed by inversion and the absorbance at 340 nm was read after 2 min incubation at ambient temperature. The results were read against calibration curve prepared for Leu-Gly solution and expressed in mM Gly/100 g of cheese sample.

### 4.4. Study Arrangement and Statistical Analysis

The whole procedure of cheese production was repeated twice for each milk type (two series) and the samples in each series were analyzed in duplicates (*n* = 4). Basic chemical composition and fatty acid profile was assessed only on fresh cheese samples, whereas microbiological studies, pH, water activity, antioxidant capacity and proteolysis both on fresh and stored for two weeks at 4 °C cheese samples. Obtained results were statistically analyzed with *t*-test (raw milk composition given in Table 1), two-way (analyzes performed only on fresh samples) or three-way ANOVA using Statistica 13.3 (TIBCO Software Inc., Palo Alto, CA, USA) software. Type of milk source (i.e., PHF and PR), cheese type (natural/plain without herbs and herbal with 0.5% bear garlic addition) and time of storage (0 week—fresh cheese, 2 week—stored cheese) were the variability factors. Significance of differences between the average values, when applicable, was estimated using Tukey post hoc test.

## 5. Conclusions

On the basis of the conducted study it was shown that cheeses produced from the milk of the native Polish Red cow breed (PR) are a better source of n-3 PUFA in a diet, and are characterized as more favorable from a nutritional point of view n-6/n-3 fatty acid ratio than cheeses produced from milk delivered from Polish Holstein-Friesian cows (PHF). Bear garlic addition did not influence the level of total or starter bacteria, yeasts and molds. However, cheeses produced from PR milk and supplemented with bear garlic leaves were characterized by a higher proteolysis degree than other treatments. All cheese samples were also free from coliforms contamination. Only a slight increase in total phenolic content and antioxidant properties measured as DPPH radical scavenging activity was observed upon bear garlic supplementation. One can suggest that 0.5% addition of herbs may be too low to observe more significant effects on the antioxidant properties in cheese. Moreover, a different preservation method or utilization of fresh plant material may improve these characteristics. The results of the conducted research indicate that bear garlic leaves can be used as a valuable additive to soft rennet cheese, favorably to those produced from milk of Polish Red cow breed, but further studies are needed to assess sensory acceptance and the other properties of novel functional cheeses.

## Figures and Tables

**Table 1:** Basic chemical composition of milk used for cheese production (g/100 g, average ± SE, *n* = 4).

Milk Source	TS	Lactose *	Fat	Protein	Ash
PHF	12.00 ^a^ ± 0.06	4.24 ^a^ ± 0.12	4.05 ^a^ ± 0.05	3.05 ^a^ ± 0.00	0.63 ^a^ ± 0.03
PR	12.72 ^b^ ± 0.13	4.05 ^a^ ± 0.05	4.53 ^a^ ± 0.12	3.47 ^b^ ± 0.06	0.73 ^b^ ± 0.01

PHF—Polish Holstein-Friesian cow breed, PR—Polish Red cow breed, T—total solids, * lactose content was calculated by the following subtraction: lactose = TS—fat—protein—ash, ^ab^ Different letters denote statistically significant differences between mean values at *p* ≤ 0.05.

**Table 2:** Chemical composition of fresh cheeses (g/100 g if not stated otherwise, average ± SE, *n* = 4).

Variability Factor		TS	Fat		Protein	Ash	NaCl
			(g/100 g)	(g/100 g TS)			
PR	N	41.80 ^a^ ± 1.17	22.30 ^a^ ± 0.83	53.36 ^a^ ± 1.48	18.48 ^a^ ± 1.13	2.2275 ^a^ ± 0.0225	0.48 ^a^ ± 0.02
	BG	48.53 ^bc^ ± 1.52	22.75 ^a^ ± 1.39	46.88 ^a^ ± 2.45	21.23 ^ab^ ± 0.76	2.5675 ^a^ ± 0.1146	0.43 ^a^ ± 0.10
PHF	N	46.48 ^b^ ± 0.63	24.75 ^a^ ± 1.05	53.35 ^a^ ± 2.86	18.78 ^a^ ± 0.80	2.3175 ^a^ ± 0.1368	0.63 ^a^ ± 0.07
	BG	52.84 ^c^ ± 0.93	26.38 ^a^ ± 1.25	50.05 ^a^ ± 3.04	23.35 ^b^ ± 0.97	2.6250 ^a^ ± 0.1574	0.65 ^a^ ± 0.05
MS		*	*	ns	ns	ns	*
ChT		*	ns	ns	*	*	ns
MS × ChT		ns	ns	ns	ns	ns	ns

MS—milk source: PR—milk from Polish Red cows; PHF—milk from Polish Holstein-Friesian cows; ChT—cheese type: N—natural cheese (without additives), BG—cheese with herbs (bear garlic), TS—total solids; ^abc^ Different letters in a column denote statistically significant differences between the mean values at *p* ≤ 0.05, * denotes statistically significant effect at *p* ≤ 0.05, ns—denotes statistically not significant effect (*p* > 0.05).

**Table 3:** Fatty acid profile (%) in the analyzed fresh cheeses (average ± SE, *n* = 4).

	PR		PHF		Effect		
	N	BG	N	BG	MS	ChT	MS × ChT
C4:0	2.12 ^a^ ± 0.01	1.83 ^a^ ± 0.12	1.62 ^a^ ± 0.54	1.96 ^a^ ± 0.05	ns	ns	ns
C6:0	1.53 ^b^ ± 0.05	1.33 ^ab^ ± 0.08	1.20 ^a^ ± 0.05	1.11 ^a^ ± 0.03	*	*	ns
C8:0	1.01 ^b^ ± 0.04	0.90 ^b^ ± 0.05	0.66 ^a^ ± 0.02	0.63 ^a^ ± 0.01	*	ns	ns
C10:0	2.29 ^b^ ± 0.11	2.09 ^b^ ± 0.11	1.30 ^a^ ± 0.03	1.26 ^a^ ± 0.01	*	ns	ns
C12:0	2.75 ^b^ ± 0.13	2.61 ^b^ ± 0.09	1.53 ^a^ ± 0.02	1.53 ^a^ ± 0.02	*	ns	ns
C13:0	0.08 ^b^ ± 0.00	0.07 ^b^ ± 0.01	0.06 ^ab^ ± 0.00	0.05 ^a^ ± 0.01	*	*	ns
C14:0	10.18 ^b^ ± 0.19	9.95 ^b^ ± 0.10	6.74 ^a^ ± 0.03	6.75 ^a^ ± 0.10	*	ns	ns
C15:0	1.27 ^a^ ± 0.01	1.26 ^a^ ± 0.00	1.08 ^a^ ± 0.05	1.10 ^a^ ± 0.06	*	ns	ns
C16:0	28.92 ^a^ ± 0.43	29.39 ^a^ ± 0.32	28.41 ^a^ ± 0.78	28.63 ^a^ ± 0.59	ns	ns	ns
C17:0	0.68 ^a^ ± 0.01	0.70 ^a^ ± 0.02	0.66 ^a^ ± 0.02	0.63 ^a^ ± 0.03	ns	ns	ns
C18:0	12.86 ^a^ ± 0.29	13.29 ^a^ ± 0.14	14.38 ^b^ ± 0.25	14.53 ^b^ ± 0.26	*	ns	ns
C20:0	2.07 ^a^ ± 0.20	1.60 ^a^ ± 0.50	1.44 ^a^ ± 0.16	1.43 ^a^ ± 0.14	ns	ns	ns
Σ SFA	65.85 ^b^ ± 0.09	65.04 ^b^ ± 0.57	59.08 ^a^ ± 0.71	59.60 ^a^ ± 0.59	*	ns	ns
C10:1	0.26 ^b^ ± 0.01	0.23 ^b^ ± 0.01	0.14 ^a^ ± 0.00	0.13 ^a^ ± 0.01	*	ns	ns
C14:1	0.61 ^a^ ± 0.02	0.85 ^b^ ± 0.00	0.56 ^a^ ± 0.05	0.59 ^a^ ± 0.02	*	*	*
C16:1 (n-9)	0.29 ^a^ ± 0.01	0.26 ^a^ ± 0.00	0.24 ^a^ ± 0.02	0.18 ^a^ ± 0.06	ns	ns	ns
C16:1 (n-7)	1.82 ^a^ ± 0.06	1.86 ^a^ ± 0.05	1.90 ^a^ ± 0.01	1.88 ^a^ ± 0.01	ns	ns	ns
C17:1	0.22 ^a^ ± 0.01	0.22 ^a^ ± 0.00	0.32 ^a^ ± 0.06	0.26 ^a^ ± 0.02	ns	ns	ns
C18:1 (cis-11)	6.57 ^b^ ± 1.30	5.12 ^ab^ ± 0.46	3.25 ^a^ ± 0.57	3.96 ^ab^ ± 0.55	*	ns	ns
C18:1 (n-9)	21.26 ^a^ ± 1.34	22.92 ^a^ ± 0.63	31.71 ^b^ ± 0.52	30.73 ^b^ ± 0.35	*	ns	ns
Σ MUFA	30.95 ^a^ ± 0.10	31.45 ^a^ ± 0.23	38.12 ^b^ ± 0.40	37.74 ^b^ ± 0.34	*	ns	ns
C18:2 (trans-9,12; n-6)	0.31 ^b^ ± 0.04	0.22 ^b^ ± 0.02	0.07 ^a^ ± 0.01	0.09 ^a^ ± 0.03	*	ns	ns
C18:2 (n-6)	1.49 ^a^ ± 0.03	1.53 ^a^ ± 0.03	2.03 ^b^ ± 0.13	1.84 ^ab^ ± 0.09	*	ns	ns
C18:3 (n-6)	0.00 ^a^ ± 0.00	0.35 ^a^ ± 0.35	0.02 ^a^ ± 0.01	0.09 ^a^ ± 0.08	ns	ns	ns
C18:3 (n-3)	1.21 ^b^ ± 0.05	1.33 ^b^ ± 0.12	0.47 ^a^ ± 0.14	0.64 ^a^ ± 0.11	*	ns	ns
n-6/n-3	1.49 ^a^ ± 0.06	1.55 ^a^ ± 0.11	5.73 ^b^ ± 1.40	3.53 ^b^ ± 0.56	*	ns	ns
CLA	0.13 ^a^ ± 0.01	0.10 ^a^ ± 0.04	0.18 ^a^ ± 0.03	0.15 ^a^ ± 0.03	ns	ns	ns
Σ PUFA	3.14 ^a^ ± 0.04	3.52 ^a^ ± 0.43	2.78 ^a^ ± 0.31	2.65 ^a^ ± 0.27	ns	ns	ns

MS—milk source: PR—milk from Polish Red cows, PHF—milk from Polish Holstein-Friesian cows, ChT—cheese type: N—natural cheese without herbs, BG—cheese with bear garlic; */ns—statistically significant/not significant; ^ab^ Different letters denote statistically significant differences between mean values at *p* ≤ 0.05.

**Table 4:** Microbiological quality of the analyzed cheeses (average ± SE).

	Cheese Type:		N		BG		Statistical Effects
	Storage Time:		0 w	2 w	0 w	2 w	
TABC (log cfu/g)	Milk type:	PR	9.26 ^b^ ± 0.07	8.64 ^ab^ ± 0.22	9.21 ^b^ ± 0.10	9.05 ^ab^ ± 0.02	MS—*; ChT—ns; S—* MS × ChT—ns MS × S—ns ChT × S—* MS × ChT × S—ns
		PHF	8.90 ^ab^ ± 0.13	8.33 ^a^ ± 0.27	8.72 ^ab^ ± 0.09	8.67 ^ab^ ± 0.05	
*Lactococcus* (log cfu/g)	Milk type:	PR	9.00 ^a^ ± 0.01	8.48 ^a^ ± 0.15	8.92 ^a^ ± 0.04	8.78 ^a^ ± 0.06	MS—*; ChT—ns; S—ns MS × ChT—ns MS × S—ns ChT × S—ns MS × ChT × S—ns
		PHF	8.31 ^a^ ± 0.59	7.79 ^a^ ± 0.15	8.09 ^a^ ± 0.53	8.39 ^a^ ± 0.08	
Yeasts (log cfu/g)	Milk type:	PR	0.00 ^a^ ± 0.00	4.22 ^a^ ± 0.93	0.00 ^a^ ± 0.00	1.22 ^a^ ± 1.22	MS—ns; ChT—ns; S—ns MS × ChT—ns MS × S—ns ChT × S—ns MS × ChT × S—ns
		PHF	2.37 ^a^ ± 1.13	3.32 ^a^ ± 0.17	1.52 ^a^ ± 1.52	1.95 ^a^ ± 1.95	
pH	Milk type:	PR	4.66 ^a^ ± 0.05	4.51 ^a^ ± 0.02	4.52 ^a^ ± 0.01	4.60 ^a^ ± 0.03	MS—ns; ChT—ns; S—ns MS × ChT—ns MS × S—ns ChT × S—ns MS × ChT × S—*
		PHF	4.49 ^a^ ± 0.10	4.78 ^a^ ± 0.19	4.62 ^a^ ± 0.05	4.43 ^a^ ± 0.10	
Water activity	Milk type:	PR	0.924 ^a^ ± 0.002	0.951 ^a^ ± 0.015	0.932 ^a^ ± 0.011	0.946 ^a^ ± 0.021	MS—ns; ChT—ns; S—ns MS × ChT—ns MS × S—ns ChT × S—ns MS × ChT × S—ns
		PHF	0.945 ^a^ ± 0.013	0.944 ^a^ ± 0.010	0.938 ^a^ ± 0.010	0.938 ^a^ ± 0.011	

TABC—total aerobic bacteria count; MS—milk source (PR—Polish Red or PHF—Polish Holstein-Friesian cows); ChT—cheese type (N—natural, BG—with bear garlic); S—storage time (0 w—fresh/at 0 week, 2 w—stored/at 2nd week); ^ab^ Different letters denote statistically significant differences between mean values; */ns—statistically significant/not significant effect at *p* ≤ 0.05.

**Table 5:** Free amino acid content (FAA) and OPA values in the analyzed fresh and stored cheeses (average ± SE, *n* = 4).

	PR				PHF			
FAA	N		BG		N		BG	
(mg/kg)	0 w	2 w	0 w	2 w	0 w	2 w	0 w	2 w
ASP	11.12 ^a^ ± 2.92	15.94 ^ab^ ± 4.92	12.88 ^ab^ ± 3.80	16.14 ^ab^ ± 3.35	18.35 ^ab^ ± 4.47	26.51 ^abc^ ± 7.01	36.77 ^bc^ ± 7.28	46.35 ^c^ ± 6.52
SER	2.00 ^a^ ± 0.41	3.08 ^a^ ± 0.86	5.10 ^a^ ± 1.72	11.17 ^a^ ± 1.33	4.99 ^a^ ± 2.39	6.88 ^a^ ± 2.87	21.29 ^ab^ ± 9.38	32.29 ^b^ ± 6.66
GLU	31.10 ^a^ ± 4.49	40.30 ^a^ ± 7.83	46.60 ^a^ ± 11.42	57.90 ^ab^ ± 7.63	92.30 ^ab^ ± 10.00	157.80 ^bc^ ± 36.80	160.40 ^bc^ ± 30.69	201.80 ^c^ ± 41.12
GLY	0.48 ^a^ ± 0.04	0.58 ^a^ ± 0.03	1.34 ^a^ ± 0.19	2.58 ^ab^ ± 0.51	3.08 ^ab^ ± 0.80	4.37 ^ab^ ± 0.76	7.10 ^b^ ± 2.29	12.88 ^c^ ± 1.14
HIS	2.25 ^a^ ± 0.28	3.25 ^a^ ± 0.62	5.47 ^a^ ± 1.29	15.18 ^a^ ± 2.76	6.59 ^a^ ± 1.45	7.81 ^a^ ± 1.13	34.03 ^ab^ ± 9.51	57.02 ^b^ ± 22.21
ARG	1.62 ^a^ ± 0.31	2.25 ^a^ ± 0.49	4.35 ^a^ ± 1.32	11.60 ^ab^ ± 1.82	7.78 ^a^ ± 1.02	19.14 ^ab^ ± 6.34	26.00 ^ab^ ± 10.94	45.17 ^b^ ± 17.82
THR	16.81 ^a^ ± 4.06	23.69 ^ab^ ± 6.70	12.73 ^a^ ± 3.62	16.84 ^a^ ± 2.65	14.57 ^a^ ± 1.31	28.52 ^ab^ ± 3.06	29.74 ^ab^ ± 5.19	43.52 ^b^ ± 7.38
PRO	52.03 ^a^ ± 11.54	68.18 ^a^ ± 16.22	49.89 ^a^ ± 5.30	62.39 ^a^ ± 8.54	63.48 ^a^ ± 2.25	77.68 ^a^ ± 2.78	50.72 ^a^ ± 9.99	84.77 ^a^ ± 3.21
ALA	7.66 ^a^ ± 0.74	10.95 ^ab^ ± 1.76	11.55 ^ab^ ± 2.95	14.05 ^abc^ ± 2.36	10.21 ^ab^ ± 2.83	21.24 ^abc^ ± 7.14	34.16 ^bc^ ± 7.31	39.52 ^c^ ± 10.45
TYR	11.31 ^a^ ± 1.54	15.54 ^ab^ ± 2.81	18.42 ^ab^ ± 3.79	26.88 ^ab^ ± 3.64	27.12 ^ab^ ± 8.05	48.35 ^ab^ ± 18.48	62.12 ^ab^ ± 20.79	73.93 ^b^ ± 20.65
VAL	5.33 ^a^ ± 0.84	7.06 ^a^ ± 1.38	18.71 ^ab^ ± 3.42	23.38 ^ab^ ± 3.10	11.71 ^a^ ± 2.15	17.60 ^ab^ ± 4.00	56.97 ^bc^ ± 13.62	67.61 ^c^ ± 19.80
MET	0.86 ^a^ ± 0.08	1.21 ^a^ ± 0.17	4.68 ^a^ ± 1.71	10.45 ^a^ ± 2.29	3.72 ^a^ ± 0.80	7.07 ^a^ ± 1.75	42.62 ^b^ ± 7.46	50.83 ^b^ ± 7.18
LYS	18.70 ^a^ ± 5.81	26.60 ^ab^ ± 9.32	31.40 ^ab^ ± 10.91	46.20 ^ab^ ± 11.49	47.92 ^ab^ ± 5.48	66.96 ^ab^ ± 7.08	113.24 ^bc^ ± 26.90	156.56 ^c^ ± 41.62
ILE	2.15 ^a^ ± 0.32	3.00 ^a^ ± 0.55	12.63 ^a^ ± 3.86	16.63 ^a^ ± 2.40	9.21 ^a^ ± 3.41	17.69 ^a^ ± 6.74	50.29 ^b^ ± 10.05	64.73 ^b^ ± 10.69
LEU	4.06 ^a^ ± 0.71	5.60 ^a^ ± 1.08	25.50 ^a^ ± 6.27	50.78 ^ab^ ± 9.31	15.72 ^a^ ± 4.75	24.79 ^a^ ± 6.49	91.54 ^ab^ ± 28.89	132.80 ^b^ ± 42.06
PHE	3.66 ^a^ ± 0.60	4.99 ^a^ ± 0.77	17.91 ^ab^ ± 2.77	38.75 ^b^ ± 4.35	20.01 ^ab^ ± 5.65	28.38 ^ab^ ± 7.51	16.94 ^ab^ ± 6.52	29.91 ^ab^ ± 12.56
CYS	1.00 ^a^ ± 0.11	1.17 ^a^ ± 0.12	2.03 ^a^ ± 0.63	3.36 ^b^ ± 1.36	nd	nd	nd	nd
Total FAA	172.11 ^a^ ± 33.44	232.79 ^a^ ± 53.09	281.15 ^ab^ ± 62.62	424.27 ^ab^ ± 51.43	356.80 ^ab^ ± 44.43	560.83 ^ab^ ± 101.54	825.24 ^bc^ ± 177.55	1145.93 ^c^ ± 240.70
OPA(mM GLY/g)	4.89 ^a^ ± 2.34	7.16 ^a^ ± 3.42	8.22 ^a^ ± 0.55	18.75 ^b^ ± 1.01	9.39 ^a^ ± 0.66	23.80 ^b^ ± 1.27	10.19 ^a^ ± 0.03	32.19 ^c^ ± 1.09

PR—milk from Polish Red cows, PHF—milk from Polish Holstein-Friesian cows, N—natural cheese without herbs, BG—cheese with bear garlic, FAA—Free amino acids; OPA—0 w—fresh cheese at week 0, 2 w—stored cheese at week 2; nd—not detected; ^abc^ Different letters denote statistically significant differences between means at *p* ≤ 0.05.

**Table 6:** Antioxidant activity of the analyzed cheeses (in 100 g of cheese, average ± SE, *n* = 4).

	Cheese Type:		N		BG		Statistical Effects
	Storage Time:		0 w	2 w	0 w	2 w	
TPC (mg GAE)	Milk type:	PR	48.00 ^ab^ ± 8.27	45.95 ^ab^ ± 8.22	95.88 ^ab^ ± 21.93	82.01 ^ab^ ± 17.87	MS—ns; ChT—*; S—ns MS × ChT—ns MS × S—ns ChT × S—ns MS × ChT × S—ns
		PHF	26.34 ^a^ ± 10.18	17.52 ^ab^ ± 4.41	67.71 ^ab^ ± 22.75	124.80 ^b^ ± 40.95	
ARP (mM TE)	Milk type:	PR	0.012 ^a^ ± 0.001	0.025 ^abc^ ± 0.003	0.019 ^ab^ ± 0.003	0.038 ^c^ ± 0.003	MS—ns; ChT—*; S—* MS × ChT—ns MS × S—* ChT × S—ns MS × ChT × S—ns
		PHF	0.017 ^ab^ ± 0.003	0.016 ^ab^ ± 0.003	0.029 ^bc^ ± 0.003	0.023 ^ab^ ± 0.005	
FRAP (mM Fe^2+^)	Milk type:	PR	0.038 ^a^ ± 0.001	0.035 ^a^ ± 0.003	0.051 ^a^ ± 0.003	0.048 ^a^ ± 0.006	MS—*; ChT—ns; S—ns MS × ChT—ns MS × S—ns ChT × S—ns MS × ChT × S—ns
		PHF	0.079 ^a^ ± 0.052	0.111 ^a^ ± 0.054	0.156 ^a^ ± 0.088	0.167 ^a^ ± 0.076	

TPC—total phenolic content; GAE—gallic acid equivalent; TE—trolox equivalent; MS—milk source (PR—Polish Red or PHF—Polish Holstein-Friesian cows); ChT—cheese type (N—natural, BG—with bear garlic); S—storage time (0 w—fresh/at 0 week, 2 w—stored/at 2nd week); ^abc^ Different letters denote statistically significant differences between means; */ns—differences statistically significant/not significant at *p* ≤ 0.05.

## Data Availability

The data presented in this study are available on request from the corresponding author.

## Supplementary Materials

The following supporting information can be downloaded at: https://www.mdpi.com/article/10.3390/molecules27248930/s1, Table S1: Statistical effects from the ANOVA of free amino acid content (FAA) and OPA values in the analyzed fresh and stored cheeses.

## Sample Availability

Samples of the compounds are not available from the authors.

## References

[1] Domagała J., Najgebauer-Lejko D., Walczycka M., Hernik J., Walczycka M., Sankowski E., Harris B.J. (2022). Traditional unfermented and fermented liquid milk products from the Malopolska Region. Cultural Heritage—Possibilities for Land-Centered Societal Development. Environmental History.

[2] Akan E., Yerlikaya O., Akpinar A., Karagozlu C., Kinik O., Uysal H.R. (2021). The effect of various herbs and packaging material on antioxidant activity and colour parameters of whey (Lor) cheese. Int. J. Dairy Technol..

[3] Dupas C., Métoyer B., El Hatmi H., Adt I., Mahgoub S.A., Dumas E. (2020). Plants: A natural solution to enhance raw milk cheese preservation?. Food Res. Int..

[4] Güler Z. (2014). Profiles of organic acid and volatile compounds in acid-type cheeses containing herbs and spices (Surk cheese). Int. J. Food Prop..

[5] El-Sayed S.M., Youssef A.M. (2019). Potential application of herbs and spices and their effects in functional dairy products. Heliyon.

[6] Shan B., Cai Y.Z., Brooks J.D., Corke H. (2011). Potential application of spice and herb extracts as natural preservatives in cheese. J. Med. Food.

[7] Lachowicz S., Kolniak-Ostek J., Oszmiański J., Wiśniewski R. (2017). Comparison of phenolic content and antioxidant capacity of bear garlic (*Allium ursinum* L.) in different maturity stages. J. Food Process. Preserv..

[8] Zięć G., Topolska K., Łukasiewicz M., Hernik J., Król K., Prus B., Walczycka M., Kao R. (2021). North Carpathian herbs. Properties and application in food and folk medicine. Indicators of Change in Cultural Heritage.

[9] Sobolewska D., Podolak I., Makowska-Wąs J. (2015). *Allium ursinum*: Botanical, phytochemical and pharmacological overview. Phytochem. Rev..

[10] Štajner D., Popović B.M., Čanadanović-Brunet J., Štajner M. (2008). Antioxidant and scavenger activities of *Allium ursinum*. Fitoterapia.

[11] MRiRW Ser Podpuszczkowy z Leśną Nutą. https://www.gov.pl/web/rolnictwo/ser-podpuszczkowy-z-lesna-nuta.

[12] Gębczyński P., Bernaś E., Słupski J., Hernik J., Walczycka M., Sankowski E., Harris B.J. (2022). Usage of wild-growing plants as foodstuff. Cultural Heritage—Possibilities for Land-Centered Societal Development.

[13] Hanus P., Znamirowska A., Kuźniar P., Szala M., Kropiwiec K. (2016). Zastosowanie dodatku jęczmienia (*Hordeum vulgare*) i czosnku niedźwiedziego (*Allium ursinum*) w technologii kefirów z mleka koziego (in Polish). Przegląd wybranych zagadnień z zakresu przemysłu spożywczego. Monografia.

[14] Coşkun H., Tunçtürk Y. (2000). The effect of *Allium* sp. on the extension of lipolysis and proteolysis in Van herby cheese during maturation. Nahrung.

[15] Sagdiç O., Simsek B., Küçüköner E. (2003). Microbiological and physicochemical characteristics of Van herby cheese, a traditional Turkish dairy product. Milchwissenschaft.

[16] Tarakçi Z., Coşkun H., Tunçtürk Y. (2004). Some properties of fresh and ripened herby cheese, a traditional variety produced in Turkey. Food Technol. Biotechnol..

[17] Çelik S.E., Özyürek M., Altun M., Bektaşoğlu B., Güçlü K., Berker K.I., Ӧzgӧkҫe F., Apak’ R. (2008). Antioxidant capacities of herbal plants used in the manufacture of Van herby cheese: ‘Otlu peynir’. Int. J. Food Prop..

[18] Yerlikaya O., Akan E., Bayram O.Y., Karaman A.D., Kinik O. (2021). The impact of medicinal and aromatic plant addition on antioxidant, total phenolic, antimicrobial activities, and microbiological quality of Mozzarella cheese. Int. Food Res. J..

[19] Kose S., Ocak E. (2020). Determination of antioxidant and antimicrobial activity of Herby cheese. J. Food Process. Preserv..

[20] Bonanno A., Tornambè G., Bellina V., De Pasquale C., Mazza F., Maniaci G., Di Grigoli A. (2013). Effect of farming system and cheesemaking technology on the physicochemical characteristics, fatty acid profile, and sensory properties of Caciocavallo Palermitano cheese. J. Dairy Sci..

[21] Litwinczuk Z., Barlowska J., Chabuz W., Brodziak A. (2012). Nutritional value and technological suitability of milk from cows of three Polish breeds included in the genetic resources conservation programme. Ann. Anim. Sci..

[22] Simopoulos A.P. (2016). An increase in the omega-6/omega-3 fatty acid ratio increases the risk for obesity. Nutrients.

[23] Znamirowska A., Szajnar K., Rożek P., Kalicka D., Kuźniar P., Hanus P., Kotula K., Obirek M., Kluz M. (2017). Effect of addition of wild garlic (*Allium ursinum*) on the quality of kefirs from sheep’s milk. Acta Sci. Pol. Technol. Aliment..

[24] Mehdizadeh T., Razavi M., Esmaeili Koutamehr M. (2019). The effect of wild leek (*Allium ampeloprasum*) on growth and survival of Lactobacillus acidophilus and sensory properties in Iranian white cheese. Res. Innov. Food Sci. Technol..

[25] Coskun H. (1998). Microbiological and biochemical changes in herby cheese during ripening. Nahrung.

[26] Gliguem H., Ben Hassine D., Ben Haj Said L., Ben Tekaya I., Rahmani R., Bellagha S. (2021). Supplementation of double cream cheese with *Allium roseum*: Effects on quality improvement and shelf-life extension. Foods.

[27] Ivanova A., Mikhova B., Najdenski H., Tsvetkova I., Kostova I. (2009). Chemical composition and antimicrobial activity of wild garlic *Allium ursinum* of Bulgarian origin. Nat. Prod. Commun..

[28] Dżugan M., Kordiaka R., Kočániová M., Wesołowska M., Tarko T., Duda-Chodak A., Witczak M., Najgebauer-Lejko D. (2014). Czosnek niedźwiedzi (*Allium ursinum*) jako uzupełnienie wiosennej diety (in Polish). Właściwości produktów i surowców żywnościowych. Wybrane zagadnienia.

[29] Stanisavljević N., Soković Bajić S., Jovanović Ž., Matić I., Tolinački M., Popović D., Popović N., Terzić-Vidojević A., Golić N., Beškoski V. (2020). Antioxidant and antiproliferative activity of *Allium ursinum* and their associated microbiota during simulated in vitro digestion in the presence of food matrix. Front. Microbiol..

[30] Church F.C., Swaisgood H.E., Porter D.H., Catignani G.L. (1983). Spectrophotometric assay using o-phthaldialdehyde for determination of proteolysis in milk and isolated milk proteins. J. Dairy Sci..

[31] Law B.A. (1997). Microbiology and Biochemistry of Cheese and Fermented Milk.

[32] Kato H., Rhue M.R., Nishimura T., Teranishi R., Buttery R.G., Shahidi F. (1989). Role of free amino acids and peptides in food taste. Flavor Chemistry.

[33] Tarakci Z., Temiz H. (2009). A review of the chemical, biochemical and antimicrobial aspects of Turkish Otlu (herby) cheese. Int. J. Dairy Technol..

[34] Tkaczewska J., Borawska-Dziadkiewicz J., Kulawik P., Duda I., Morawska M., Mickowska B. (2020). The effects of hydrolysis condition on the antioxidant activity of protein hydrolysate from *Cyprinus carpio* skin gelatin. LWT.

[35] Wallace J.M., Fox P.F. (1997). Effect of adding free amino acids to Cheddar cheese curd on proteolysis, flavour and texture development. Int. Dairy J..

[36] Mangia N.P., Murgia M.A., Garau G., Sanna M.G., Deiana P. (2008). Influence of selected lab cultures on the evolution of free amino acids, free fatty acids and Fiore Sardo cheese microflora during the ripening. Food Microbiol..

[37] Madrau M.A., Mangia N.P., Murgia M.A., Sanna M.G., Garau G., Leccis L., Caredda M., Deiana P. (2006). Employment of autochthonous microflora in Pecorino Sardo cheese manufacturing and evolution of physicochemical parameters during ripening. Int. Dairy J..

[38] Rulikowska A., Kilcawley K.N., Doolan I.A., Alonso-Gomez M., Nongonierma A.B., Hannon J.A., Wilkinson M.G. (2013). The impact of reduced sodium chloride content on Cheddar cheese quality. Int. Dairy J..

[39] Gođevac D., Vujisić L., Mojović M., Ignjatović A., Spasojević I., Vajs V. (2008). Evaluation of antioxidant capacity of *Allium ursinum* L. volatile oil and its effect on membrane fluidity. Food Chem..

[40] Krivokapic M., Bradic J., Petkovic A., Popovic M. (2018). Phytochemical and pharmacological properties of *Allium ursinum*. Serb. J. Exp. Clin. Res..

[41] Najgebauer-Lejko D., Sady M., Grega T., Walczycka M. (2011). The impact of tea supplementation on microflora, pH and antioxidant capacity of yoghurt. Int. Dairy J..

[42] AOAC, Horwitz W., Latimer G. (2007). Official Methods of Analysis of AOAC International.

[43] (2008). Cheese—Determination of Fat Content—Van Gulik Method.

[44] (2006). Cheese and Processed Cheese Products—Determination of Chloride Content—Potentiometric Titration Method.

[45] Folch J., Lees M., Stanley G.H.S. (1957). A simple method for the isolation and purification of total lipides from animal tissues. J. Biol. Chem..

[46] Ledoux M., Chardigny J.M., Darbois M., Soustre Y., Sébédio J.L., Laloux L. (2005). Fatty acid composition of French butters, with special emphasis on conjugated linoleic acid (CLA) isomers. J. Food Compos. Anal..

[47] Moldovan B., Iasko B., David L. (2016). Antioxidant activity and total phenolic content of some commercial fruit-flavoured yogurts. Stu. U. Babes-Bol. Chem..

[48] (2010). Microbiology of Food and Animal Feeding Stuffs—Preparation of Test Samples, Initial Suspension and Decimal Dilutions for Microbiological Examination—Part 5: Specific Rules for the Preparation of Milk and Milk Products.

[49] (2013). Microbiology of the Food Chain—Horizontal Method for the Enumeration of Microorganisms—Part 1: Colony Count at 30 Degrees C by the Pour Plate Technique.

[50] (1997). Dairy Starter Cultures of Lactic Acid Bacteria (LAB).

[51] (2007). Microbiology of Food and Animal Feeding Stuffs—Horizontal Method for the Enumeration of Yeasts and Moulds—Part 2: Colony Count Technique in Products with Water Activity Less than or Equal to 0.95.

[52] (2007). Microbiology of Food and Animal Feeding Stuffs—Horizontal Method for the Enumeration of Coliforms—Colony-Count Technique.

[53] (2017). Foodstuffs—Determination of Water Activity.

