# Supplementary material for: Effect of Bear Garlic Addition on the Chemical Composition, Microbiological Quality, Antioxidant Capacity, and Degree of Proteolysis in Soft Rennet Cheeses Produced from Milk of Polish Red and Polish Holstein-Friesian Cows
Source: Molecules. 2022 Dec 15;27(24):8930. doi: 10.3390/molecules27248930 (PMC9787386; doi:10.3390/molecules27248930)
Supplement: Supplementary file 1 [file molecules-27-08930-s001.zip › molecules-2053412-supplementary.pdf]

Table S1: Statistical effects from the ANOVA of free amino acid content (FAA) and OPA values in the analyzed fresh and stored cheeses.

FAA/OPA	MS	ChT	S	MS x ChT	MS x S	ChT x S	MS x ChT x S
ASP	*	*	ns	*	ns	ns	ns
SER	*	*	ns	*	ns	ns	ns
GLU	*	*	ns	ns	ns	ns	ns
GLY	*	*	*	*	ns	ns	ns
HIS	*	*	ns	*	ns	ns	ns
ARG	*	*	ns	ns	ns	ns	ns
THR	*	ns	*	*	ns	ns	ns
PRO	ns	ns	*	ns	ns	ns	ns
ALA	*	*	ns	*	ns	ns	ns
TYR	*	*	ns	ns	ns	ns	ns
VAL	*	*	ns	*	ns	ns	ns
MET	*	*	ns	*	ns	ns	ns
LYS	*	*	ns	*	ns	ns	ns
ILE	*	*	ns	*	ns	ns	ns
LEU	*	*	ns	*	ns	ns	ns
PHE	ns	*	*	*	ns	ns	ns
CYS	nd in PHF	*	*	ns	-	-	-
Total FAA	*	*	*	*	ns	ns	ns
OPA	*	*	*	*	ns	*	ns

* statistically significant impact at $p \leq 0.05$, ns - statistically not significant impact at $p \leq 0.05$; nd in PHF – not detected in cheeses from PHF milk; MS – milk source (PR – Polish Red or PHF – Polish Holstein-Friesian cows); ChT - cheese type (N – natural, BG – with bear garlic); S – storage time (0w – fresh/ at 0 week, 2w – stored/ at 2nd week); * - statistically significant effect at $p \leq 0.05$; ns – statistically not significant effect ($p>0.05$)
